# Cardio-Oncology Challenges in Elderly Patients

**DOI:** 10.3390/jcm14093257

**Published:** 2025-05-07

**Authors:** Ester Topa, Eliana De Rosa, Alessandra Cuomo, Francesco Curcio, Marika Rizza, Francesco Elia, Veronica Flocco, Umberto Attanasio, Martina Iengo, Francesco Fiore, Maria Cristina Luise, Grazia Arpino, Roberto Bianco, Chiara Carlomagno, Mario Giuliano, Luigi Formisano, Marco Picardi, Carminia Maria Della Corte, Floriana Morgillo, Giulia Martini, Erika Martinelli, Stefania Napolitano, Teresa Troiani, Giovanni Esposito, Antonio Cittadini, Guido Iaccarino, Giuseppe Rengo, Pasquale Abete, Valentina Mercurio, Carlo Gabriele Tocchetti

**Affiliations:** 1 Department of Translational Medical Sciences (DISMET), Federico II University, 80131 Naples, Italy; 2 Department of Clinical Medicine and Surgery, Federico II University, Via Sergio Pansini 5, 80131 Naples, Italy; 3 Interdepartmental Center of Clinical and Translational Research (CIRCET), Federico II University, 80131 Naples, Italy; 4 Department of Precision Medicine, University of Campania Luigi Vanvitelli, 80131 Naples, Italy; 5 Department of Advanced Biomedical Sciences, Federico II University, 80131 Naples, Italy; 6 Hypertension Research Center “CIRIAPA”, Federico II University, 80131 Naples, Italy; 7 Center for Basic and Clinical Immunology Research (CISI), Federico II University, 80131 Naples, Italy

**Keywords:** cardio-oncology, elderly, comprehensive geriatric assessment, cardiovascular baseline evaluation, echocardiography

## Abstract

**Background and Objectives:** Along with the ageing of the population, cancer and cardiovascular (CV) diseases more frequently coexist, complicating patients’ management. Here, we focus on elderly oncologic patients, describing clinical features and comorbidities, discussing therapeutic management CV risk factors and CV complications risen during our CV follow-up, and exploring the different items of the comprehensive geriatric assessment (CGA) and the correlation between cardiac function by means of standard 2D echocardiography and each of the CGA items. **Methods:** A total of 108 consecutive patients (mean age 73.55 ± 5.43 years old; 40.7% females) referred to our cardio-oncology unit were enrolled, and three different groups were identified: Group 1, patients naïve for oncologic treatments (mean age 73.32 ± 5.40; 33% females); Group 2, patients already on antineoplastic protocols (mean age 73.46 ± 5.09; 44.1% females); and Group 3, patients who had already completed cancer treatments (mean age 74.34 ± 6.23; 55% female). The correlation between CGA, performed in a subgroup of 62 patients (57.4%), and echocardiographic parameters was assessed. **Results:** Group 2 patients had the highest incidence of CV events (CVEs) (61.8% vs. 14.8% in Group 1, 15% in Group 3; *p* ≤ 0.001) and withdrawals from oncologic treatments (8.8% vs. none in Group 1; *p* = 0.035). Group 2 had worse 48-month survival (47.1% vs. 22.2% in Group 1, 20% in Group 3; *p* = 0.05), which was even more evident when focusing on patients who died during follow-up. When assessing echocardiographic parameters, physical activity showed an inverse correlation with the left ventricular mass index (*p* = 0.034), while the Frailty index showed a direct correlation with the E/e’ ratio (*p* = 0.005). **Conclusions:** A thorough baseline CV assessment is important in elderly oncologic patients eligible for anticancer treatment. In this population, CGA can be a simple, feasible screening tool that might help identify patients at a greater risk of developing CVEs correlating to several pivotal cardiovascular parameters.

## 1. Introduction

Ageing is characterized by an increased prevalence of several chronic and degenerative conditions, such as cardiovascular diseases (CVDs) and cancer [1,2,3,4,5]. In particular, it is estimated that around 60% of new diagnoses of cancer occur in people older than 65 years [6,7]. Both cancer and cancer treatments represent external stressors that increase vulnerability in older people and can contribute to the setting of frailty, a complex geriatric syndrome characterized by a cumulative decline of functional reserves and an increased vulnerability to stressor events leading to poor health outcomes, such as falls, delirium, disability, multi-system dysfunction, chronic disease, and higher mortality [8,9]. Furthermore, thanks to advances in the multidisciplinary management of CVDs, patients’ survival, even after major CV events (CVEs), has ameliorated significantly over the past decade [10,11,12,13,14,15,16]. On the other hand, it is well known that many antineoplastic treatments are associated with diverse CV side effects, such as cardiac dysfunction associated with anthracyclines administration, myocarditis typical of the use of immune checkpoint inhibitors, or hypertension associated with VEGF inhibitors [16].

Indeed, cancer and heart failure (HF) share many common risk factors, besides being linked from a pathophysiologic standpoint [3]. On the other hand, age itself affects clinical outcomes and the quality of life in cancer patients and implies a higher likelihood of developing cardiotoxicity [7]. Although the interest on the matter has been rising, elderly cancer patients are often under-treated and still under-represented in clinical trials; hence, strong evidence on the best management of this subset of patients is lacking, and the relationship between frailty and cancer is yet to be cleared [6,7].

The treatment of elderly patients with cancer is very complex not only due to the presence of comorbidities, the use of polypharmacotherapy, and the development of disability and geriatric syndromes but also due to the higher risk of side effects related to oncologic therapies. The clinical decision of the oncologist concerning the best treatment of an elder patient is based on an explicit estimation of the patient’s life expectancy and quality of life.

The International Society of Geriatric Oncology (SIOG) recommends the comprehensive geriatric assessment (CGA) as the gold standard to evaluate frailty in elderly cancer patients, since its use in the onco-geriatric setting has been proven to improve patients’ outcomes, quality of life, and survival [6,7]. In particular, the CGA is a multidimensional evaluation tool which investigates different domains of frailty to detect and stratify clinical vulnerability [8,17].

How CVDs can contribute to frailty in cancer patients is still unclear [11,18]. Improving the knowledge of the epidemiology and phenomenology of frailty in elderly subjects with cancer is crucial in order to develop the best effective strategy of individual care in such a heterogeneous and challenging category of patients [7]. Furthermore, it is worth mentioning that there is a tight link between ageing and cachexia, further worsening the survival of elderly cancer patients [2,8,10].

Although, to date, there is no conclusive evidence on the impact of geriatric interventions in elderly cancer patients, CGA is the indispensable prerequisite for planning patients’ clinical paths, implementing the necessary interventions to allow fragile patients to benefit from treatments.

The objectives of this work are to analyze the characteristics of elderly patients who are referred to our cardio-oncology unit, outlining clinical features and comorbidities, and to discuss complex therapeutic management for the prevention and optimization of cardiovascular risk factors, as well as the treatment of cardiovascular complications. Finally, we aim at exploring whether CGA might be a potential tool to evaluate the risk of CVEs in elderly patients and its association to other recognized clinical and echocardiographic parameters.

## 2. Materials and Methods

### 2.1. Study Design

In this analysis, we enrolled 108 consecutive elderly (age > 65 years) patients pooled from our recently published single-center prospective study performed in our cardio-oncology unit in the Department of Translational Medical Sciences, Federico II University, Naples, Italy [19,20]. The protocol was approved by the local ethics committee, and the study was conducted following the Helsinki Declaration principles, and all patients signed a written informed consent form to participate in this study. Most of the patients included in the study were enrolled consecutively in our unit from major oncology university clinics such as the Hematology and Oncology Divisions of the Department of Clinical Medicine and Surgery of the Federico II University Hospital of Naples and the Division of Oncology and the Department of Precision Medicine of Luigi Vanvitelli University of Campania, Naples, Italy. Some patients were enrolled from smaller oncology units in our region. Inclusion criteria were age > 65 years; patients with recent cancer diagnosis eligible for anticancer therapies, patients already on oncologic regimens, or patients who had formerly received antineoplastic therapies; and availability of at least two visits in our cardio-oncology unit at least 1 month apart from one another.

### 2.2. Cardio-Oncologic Assessment

The cardiovascular assessments included complete patient history, lifestyle (diet, activity, and smoking habits), family history of heart disease, any comorbidities and concomitant treatments, and previous diseases and therapies; full physical examination, blood pressure measurement, and resting 12-lead ECG; and 2D echocardiography [21] and blood tests, including biomarkers such as N-terminal pro-brain natriuretic peptide and cardiac Troponin I [22,23]. Conventional transthoracic 2D echocardiographic examinations were performed with a Philips iE33 ultrasound equipment (Phillips Healthcare, Andover, MA, USA). Images were obtained using a 3.4 MHz transducer, with subjects in the left lateral decubitus position. According to the American Society of Echocardiography (ASE) and European Association of Cardiovascular Imaging (EACVI) recommendations, standard subxiphoid, apical, and parasternal windows were visualized to acquire 2D images of the heart chambers, color, and pulsed-wave and continuous-wave Doppler measurements to assess diastolic and systolic cardiac function [24,25]. To assess heart function, we analyzed left ventricular ejection fraction (LVEF), which was visualized from the apical four-chamber and two-chamber views, with the modified Simpson’s rule [25,26].

### 2.3. Clinical Characteristics of the Study Population

As previously described [19], our population was divided into 3 clinical types according to their oncologic status at the first CV assessment:

- Group 1: subjects with recent cancer diagnosis and naïve for anticancer regimens.
- Group 2: subjects undergoing antineoplastic protocols.
- Group 3: subjects who had already finished anticancer treatments.

### 2.4. Geriatric Evaluation

A total of 62 of the 108 patients also underwent a comprehensive geriatric multidimensional assessment (CGA) which included

- The Mini-Mental State Examination (MMSE) for the evaluation of cognitive impairment [27];
  - The Geriatric Depression Scale (GDS) for the evaluation of depression [28];
  - The Cumulative Illness Rating Scale C (CIRS-C) for comorbidity [29];
  - The Cumulative Illness Rating Scale G (CIRS-G) for severity [29];
  - The number of drugs used;
  - Basic and Instrumental Activity of Daily Living (BADL and IADL) for the evaluation of autonomy [30,31];
  - A Mini Nutritional Assessment (MNA) for the evaluation of the risk of malnutrition [32];
  - The Tinetti Scale for balance and gait evaluation [33];
  - The Short Physical Performance Battery (SPPB) [34];
  - A Physical Activity Scale for the Elderly (PASE) [35];
  - A Social Support Assessment (SSA) [36];
  - The Italian Frailty Index (IFI), derived from a validated scale used in a cohort of New Haven (CT, USA) patients in the Yale Precipitating Events Project study [37] and validated by Abete and coworkers in 2017 in a population of 1077 subjects aged 65 years or older from Southern Italy [8].

These patients were then divided into two subgroups:

- Subgroup A: survived patients;
- Subgroup B: patients who died during follow-up.

### 2.5. Follow-Up Visits

Patients were assessed every 3 months until 1 year after the end of oncologic regimens, then every 6 months for 5 years, and then once a year, or when clinically needed.

### 2.6. Outcomes

Considered outcomes were (1) new CV events during follow-up, including hospitalizations for CV reasons, (2) new-onset CV events involving antineoplastic treatment temporary suspension or modification, (3) CV events involving oncologic protocol definitive suspension, and (4) death for all causes. Moreover, we also analyzed information on CV therapy optimization (also known as the modification of CV drugs already prescribed) or the de novo prescription of CV therapies. The diagnosis of new-onset CV events was discussed by our expert team of specialists in cardio-oncology. Antineoplastic treatments adjustments were collegially addressed with the referring specialist in oncology, considering benefits and risks.

We then aimed to assess frailty by CGA by comparing CGA results among the 3 aforementioned groups of patients and discriminating them according to survival, evaluating the correlation between CGA domains and cardiac function indicators, and comparing echocardiographic parameters and CGA parameters according to survival.

### 2.7. Statistical Analysis

The normality of the distribution of the continuous variables was assessed by means of the Kolmogorov–Smirnov test. The continuous variables are presented as the mean ± standard deviation (SD). The discrete variables are expressed as absolute number and relative percentage. A comparative analysis between groups was performed using ANOVA. Differences between groups were assessed by means of Student’s t test for unpaired data. Linear regression was used for the continuous variables to evaluate the correlation between cardiac function parameters, echocardiographic parameters, as independent variables, and CGA results, as dependent variables. Differences were considered statistically significant for *p*-values < 0.05. Statistical analysis was performed using the Statistical Package for Social Science Statistics Version 24 (IBM, Armonk, NY, USA). Univariate analysis was performed to assess the relationship between different parameters and the risk of developing cardiovascular events during follow-up.

Prior to enrollment, all patients provided informed consent, and the study was conducted in accordance with the principles of the Declaration of Helsinki.

## 3. Results

### 3.1. Cardiovascular Characteristics of Patients

From January 2015 to February 2020, a total of 108 subjects met the inclusion criteria and were enrolled in the study. Table 1 describes the clinical and oncologic characteristics of the patients. Group 1 patients, including subjects naïve for oncologic treatments, comprised the large part of our study population. Table 1 shows that no significant differences were found between the three groups.

### 3.2. Cancer Characteristics and Protocols

Data on cancer characteristics, including antineoplastic protocols, are presented in Table 1. In particular, 42 patients (38.9%) had colon cancer; a total of 14 patients (12.9%) had lymphomas, and 13 patients (12%) were affected by breast cancer. Data concerning other cancer sites are shown in Table 1.

Thirty-three patients (30.5%) were administered with oncologic regimens, including pyrimidine analogues and/or platinum-derived compounds, eventually in association with other anticancer drugs (i.e., irinotecan, taxanes, etoposide, and gemcitabine). A total of 26.8% of patients were administered with oncologic regimens, including endothelial growth factor receptor inhibitors and/or VEGF-I (vascular endothelial growth factor inhibitors), eventually along with different drugs (i.e., platinum-derived compounds, taxanes, irinotecan, pyrimidine analogues, gemcitabine, and etoposide). Further information about specific anticancer treatments according to the patients’ group is shown in Table 1.

### 3.3. Outcomes

**Figure 1:**
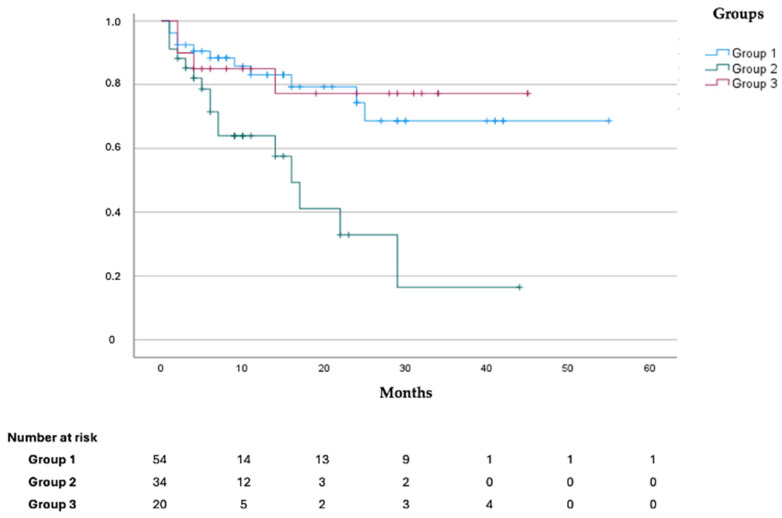
Kaplan–Meier curves for survival according to the three group of patients.

**Figure 2:**
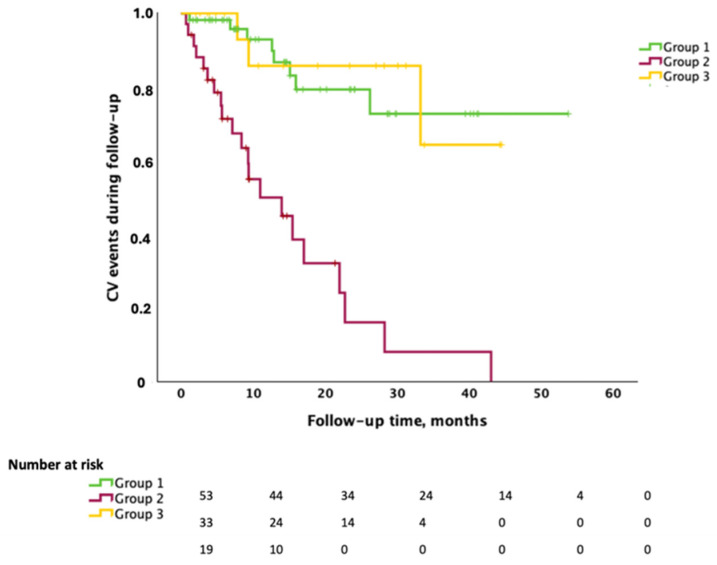
Kaplan–Meier curves for CV event incidence according to the three group of patients.

Outcomes data for the whole cohort and for each patient group are shown in Table 2. Thirty-two patients (29.6% of the total, 65.6% of whom from Group 2) presented with CV events during follow-up. Twelve patients (ten from Group 2) showed CV events of such severity that they required antineoplastic regimen temporary suspension or modification, and three patients, all part of Group 2, presented with severe CV events, leading to definitive antineoplastic treatment suspension. Among CV events presented, the most common were new-onset arterial hypertension, subclinical (increase in N-terminal pro-brain natriuretic peptide levels [23]) or clinical worsening of LV function (decrease of LVEF [21]) during the administration of antineoplastic treatments, and deep vein thrombosis during chemotherapy administration. The univariate analysis showed that Group 2 was independently associated with a higher risk of developing CV events during follow-up (OR 9.154 [2.237–37.451]; *p* = 0.002). We registered thirty-two deaths from all causes during our follow-up, of which 50% (16 patients out of 32) belonged to Group 2. The Kaplan–Meier curves stratified for the three types suggest that Group 2 patients present worse 48-month survival (*p*-value = 0.05) (Figure 1). As shown in Figure 2, Kaplan–Meier curves according to the three types suggest that Group 2 patients present a higher incidence of CV events during follow-up (log rank *p* value < 0.001).

### 3.4. Cardiovascular Events During Follow-Up and Treatment Implementations

During follow-up, 11 patients (10.1%), including 4 from Group 1, 5 from Group 2, and 2 from Group 3, were diagnosed with arterial hypertension. Four patients (3.7%), two from Group 1, one from Group 2, and one from Group 3, received a diagnosis of heart failure with reduced ejection fraction (HFrEF), and four patients (3.7%), three of whom were from Group 1 and one from Group 2, developed atrial fibrillation. Six patients (5.5%), 3 from Group 1, 1 from Group 2 and 2 from Group 3, developed deep vein thrombosis. Finally, a Group 2 patient had an acute myocardial infarction. Table 3 shows the changes in CV medications prescribed during follow-up. Indeed, numerous patients underwent an optimization of their pre-existing cardiac therapies. Nineteen subjects (17.6%) up-titrated beta-blockers. For eight patients (7.4%), we increased the dose of an angiotensin-converting enzyme inhibitor. In six patients, we up-titrated the dosage of angiotensin receptor blockers. Antiplatelet therapy was increased in three patients (2.8%). Statins were up-titrated in 13 patients (12%), as it was the dosage of diuretics in 6 patients (5.6%). The calcium channel blocker dose was increased in five patients (4.6%). For one patient (0.9%) in Group 2, the dosage of an angiotensin receptor neprilysin inhibitor (ARNI) was increased.

Additionally, 31 patients (28.7%) started beta-blockers. Seven patients (6.5%) started angiotensin-converting enzyme inhibitors. In five patients (4.6%), angiotensin receptor blockers were newly prescribed. Moreover, 33 patients (30.6%) started antiplatelet therapy, and 27 patients (25%) started statins. Furthermore, 22 patients (20.4%) started diuretics, and 8 patients (7.4%) started calcium channel blockers. Finally, 12 subjects (11.1%) started anticoagulation treatment, and 2 patients (1.9%) were prescribed with an ARNI.

### 3.5. Comprehensive Geriatric Assessment

Out of the 108 patients, 62 underwent a comprehensive geriatric assessment (CGA) at the first cardiology visit (mean age 73.55 ± 5.41 years, 38 (61.3%) men).

There were no differences in terms of clinical and echocardiographic characteristics between patients who presented and those who did not present the CGA (Table 4). Furthermore, correlation analyses between CGA variables and clinical and echocardiographic parameters were performed solely on those presenting the CGA.

According to the frailty index, our population showed mild frailty [8], but no impairment of cognitive function was assessed using MMSE, and no depression was assessed with the GDS. Furthermore, our patients showed less than one lost function among BADL and less than two lost functions among IADL, indicating a low risk of falls according to the Tinetti scale, with reduced SPPB and PASE scores. Finally, based on MNA, our population was at risk of developing malnutrition due the presence of the mild comorbidity index according to CIRS. Polypharmacotherapy was also assessed, showing an average of 6.7 ± 3.3 drugs per prescription (Table 5).

### 3.6. CGA and Echocardiographic Measurements

Concerning the CGA, patients in Group 2 showed lower mean values for the Tinetti, SPPB, PASE, and MNA scales, indicating the presence of increased risk of malnutrition, sedentary lifestyle, and low physical performance. In addition, the frailty index was higher in patients from Group 2, while the values of CIRS-G only showed a mild degree of comorbitities. The evaluation of the echocardiographic parameters shows that the mean EF of patients belonging to Group 2 is lower than that of Groups 1 and 3. The CGA results according to each group are reported in Table 5, while echocardiographic measurements are shown in Table 6.

We then further stratified our population according to survival: Subgroup A, including patients alive at the end of our follow-up, and Subgroup B, including the 19 patients (31%) who died from all causes during the study period.

Comparing the CGA results, patients in Subgroup B presented with a larger number of prescribed drugs compared to subgroup A (*p* = 0.035), while PASE was lower, which is suggestive of a sedentary lifestyle (*p* = 0.004) (Table 7). In addition, patients belonging to Subgroup B, compared to Subgroup A patients, displayed a trend towards a higher risk of malnutrition (*p* = 0.073), worsen physical performance (*p* = 0.060), and less autonomy in basic and instrumental activities of daily life (*p* = 0.074 and *p* = 0.071, respectively).

Comparing echocardiographic measurements, there was no difference in terms of EF, but Subgroup B patients presented with a thicker interventricular septum, which is with a higher LV mass (respectively *p* = 0.009 and *p* = 0.036) (Table 8).

### 3.7. Correlation Between Cardiac Function Indicators and CGA Results

Finally, physical activity evaluated using PASE showed a statistically significant inverse correlation (*p* = 0.034) with the left ventricular mass index. Patients with a sedentary lifestyle showed cardiac hypertrophy and increased diastolic filling pressures.

The frailty index showed a statistically significant direct correlation (*p* = 0.005) with E/e’ at echo.

## 4. Discussion

Along with the aging of the world population, there is an increase in the prevalence of CVDs and cancer [38,39,40,41,42]; indeed, more subjects are referred to cardio-oncology units [43,44,45,46]. Our real-world study assesses the clinical characteristics of subjects referred daily to our cardio-oncology unit from two major oncology university clinics (Federico II University and Vanvitelli University, both in Naples, Italy) and from smaller oncology units in our region, describing clinical challenges that we face with these patients. This work underlines the pivotal role of a strict cardiovascular follow-up in elderly oncologic subjects, including a meticulous baseline CV evaluation, as recommended by the 2022 ESC guidelines on cardio-oncology [20], thereby tailoring follow-ups to patients’ specific characteristics [21,23].

Data from the current work further consolidate the knowledge that an accurate baseline cardiovascular assessment is fundamental to guarantee the completion of anticancer treatment, even more so when dealing with fragile patients, such as the elderly population. In particular, as shown in the Kaplan–Meier curve, the survival of patients who are referred to our unit when already on anticancer treatments (Group 2) is significantly worse compared to the other two groups (Figure 1).

As already shown in our previous work [19], the baseline evaluation by an experienced cardio-oncology unit is pivotal for oncologic patients, and this is even more true when dealing with more fragile patients, such as patients with overt CVDs or elderly patients, as shown in the current manuscript. Furthermore, it is well known that among the antineoplastic drugs administered to our cohort of patients, there is a plethora of side effects that might impair the completion of oncologic protocols [47]. For this reason, a thorough evaluation of each patient at baseline is pivotal.

The management of oncologic elderly patients experiencing concomitant CVDs is burdened by multiple difficulties. Indeed, as for patients with overt CVDs, elderly comorbid patients are often excluded from randomized clinical trials. Furthermore, the up titration of many cardiovascular drugs might be a slow process, even more so in elderly patients that might experience more side effects compared to younger subjects.

The CGA is a clinical instrument to evaluate the functional burden of diseases. Disability, evaluated using BADL and IADL, is reported to be an important prognostic stratification tool and is more effective than multimorbidity, as it remarkably affects mortality, independently from age and other functional and clinic variables [48]. The correlation between gait speed and survival is also well known, and gait speed has been described as a simple and feasible indicator of elderly patients’ health [49]. Chiarantini and colleagues showed that physical performance, evaluated using SPPB, is an independent predictor of long-term survival in elderly subjects admitted to the hospital for heart failure [50]. In the subset of patients for whom the CGA evaluation was available, we demonstrated that patients who died during follow-up performed worse at the CGA, underlying the important role that a comprehensive multimodal evaluation including the CGA could play in elderly oncologic patients. In particular, patients who died during our follow-up displayed significant differences in several CGA scales vs. patients who survived, indicating higher numbers of administered drugs and lower physical activity (PASE).

The MNA result scores indicate a higher risk of malnutrition. These results are consistent with many studies that point out that weight loss and malnutrition are associated with dose-limiting chemotherapy toxicity and mortality in cancer patients [51], with polypharmacy being related to several clinical outcomes including adverse drug reactions, falls, frailty, hospitalization, postoperative complications, and mortality [52]. Nutrition interventions have been showed to be effective in reducing mortality in cancer patients, suggesting a key role of nutritional screening in the context of a geriatric assessment [53]. It is reported that increasing physical activity could lower cancer-related mortality, and physical activity programs during cancer treatments can be safe and feasible, reduce fatigue, limit weight gain, and increase the quality of life [54,55,56]. These findings highlight the importance of investigating physical activity level in order to include interventions targeting its improvement in the management of cancer patients.

The comorbidity indexes CIRS-C and CIRS-G were only mildly affected, but this is probably due to the fact that the CIRS scale does not include cancer among the items. Many studies describe the prognostic value of functional status and comorbidities on survival, pointing out the importance of detecting patients without irreversible or severe comorbidities who have a long life expectancy, as they are eligible to receive full treatment regimens [57,58,59,60,61]. On the other hand, the use of evaluation scales should not bring the risk of under-treating patients; a multidisciplinary approach involving geriatricians and haemato-oncologists is therefore necessary in the management of elderly cancer patients [57].

Frailty is related to worse clinical outcomes in elderly patients, particularly those with heart failure, for whom a systematic evaluation of frailty is recommended and plays a key role in the therapeutic decision-making process and the rehabilitation program [11]. In a meta-analysis involving 6896 patients, Denfeld and coworkers pointed out that heart failure, regardless of age, is associated with a higher prevalence of frailty, compared to the one observed in community-dwelling “oldest-old” subjects [11]. How cardiovascular dysfunction can contribute to frailty is still unclear. The ARIC study, conducted on 3991 older adults, aimed to clarify the relationship between frailty and cardiovascular function and assessed whether such association is independent of comorbidities, and it reported that frail patients showed a higher prevalence of abnormal cardiac parameters, compared to non-frail and prefrail subjects, and several cardiac alterations, including left ventricular hypertrophy. Reduced global longitudinal strain and higher left atrial volume index scores were independently associated with frailty. These findings allowed us to hypothesize that cardiovascular dysfunction could concur to the pathophysiology of frailty [62]. In the present study, we evaluated the correlation among several echocardiographic indicators of cardiac function and each of the CGA items. Interestingly, physical activity evaluated using PASE showed a significant inverse correlation with the left ventricular mass index, indicating that the lower the physical activity, the higher the left ventricular mass index. The frailty index showed a significant directly correlation with E/e’, a marker of ventricular stiffness.

Our analysis shows that patients with worse cardiac performance in terms of diastolic and systolic function have reduced physical activity and higher frailty index scores, characteristics of patients who died during the study.

These findings support the importance of further investigating the association between cardiopulmonary function and functional capacity, especially in older subjects, whose functional decline is part of the frailty setting as a real geriatric syndrome [9]. Interestingly, in a 2018 study conducted on 191 elderly subjects by Kusunose and coworkers, frail patients, compared to non-frail, were more likely to have larger left atrial size, lower stroke volume, and worse diastolic dysfunction, and frailty was associated with future cardiovascular events [63,64]. Our findings were consistent with these results, pointing out that the assessment of frailty using the CGA could be a simple, feasible, and low-cost screening tool to identify patients in an onco-geriatric setting who are more likely to have cardiac dysfunction [63,64,65], thereby helping to establish the priority of further investigation and/or the need for a more stringent follow-up.

As for the timing of follow-ups, considering that members of our research group actively participated in the drafting of the 2022 ESC cardio-oncology guidelines, for the present study, we applied the same suggestions available in the aforementioned document [20].

Finally, identifying the association between PASE and heart function could be worth it to implement adapted physical activity in elderly cancer patients, as it is well known that exercising regularly exerts a beneficial effect not only on mental health but also on cancer response [65,66]. Necessarily, new approaches to examine physical status and frailty in elderly patients are emerging, so it could be interesting to further investigate how and if these new tools, eventually based on artificial intelligence, and approaches might be helpful in this field [67,68].

## 5. Limitations

Among the limitations of our work, the most important is the rather small sample size, taking into account that the enrolled subjects are further stratified into smaller groups according to survival during follow-up. Such limited number of patients might negatively affect the strength of our results, and our study’s generalizability is particularly limited. Indeed, further research is needed to expand the knowledge on cardiovascular care in elderly cancer patients.

Even though our patients are mainly referred from two major oncology university clinics in Naples (and from other smaller oncology services in the Naples area), this is a cardiologic–monocentric descriptive analysis.

In addition, patients were treated with very heterogeneous therapies, and different forms of cancer were included, which complicates intergroup analysis. Unfortunately, because of the relatively small number of patients and the heterogeneity of their antineoplastic treatment, it was not possible to stratify patients according to antineoplastic protocols; thus, it was not possible to explore the specific effect of each antineoplastic agent. In the future, we aim to perform a multicenter study by expanding our approach to other cardio-oncology units to address institutional biases and manage the impact of patient population demographics on the results. This way, we may validate our results in cohorts of different patients and explore specific relationships between each oncologic protocol and CGA. In our study, the cancer type distribution does not completely overlap the cancer type distribution in Italy, as our population is mostly composed of elderly patients with colorectal cancer. This represents a limitation of the study, but on the other hand, considering the overall age of our population, this does not come completely by surprise, as colorectal cancer is one of the most common types of cancer in elderly patients. However, these preliminary data strongly suggest that more attention should be given to CGA in elderly cancer patients. Unfortunately, our patients underwent only one CGA at their first cardio-oncologic evaluation in our clinic. In the future, we aim to explore the relevance of multiple CGAs as part of elderly cancer patients’ follow-up to assess whether changes in CGA over time might be predictive of outcomes in this setting.

In addition, we only collected scattered data regarding the biochemical and bio-humoral characteristics of the patients at the start of the study, such as troponin levels or natriuretic peptides; hence, we could not correlate them to a prognosis. Finally, since none of our patients died directly because of cardiovascular diseases, it is not easy to distinguish between direct and indirect cardiovascular deaths, especially in elderly patients [69]. In particular, ageing is associated with an increased risk of cachexia and muscular wasting [69]. Keeping in mind these considerations, elderly patients present a high risk of developing CVEs due to anticancer treatment. The rate of such events is significantly lower in those subjects referred to our cardio-oncology unit before starting anticancer regimens, as there were no differences in terms of CV risk factors between subjects in Group 1 and Group 2. These results support strict follow-up in high-risk CV patients. Correcting CV risk factors and titration of therapy is fundamental for these patients before starting oncologic treatment.

## 6. Conclusions

The present study suggests that CGA can help detect non-frail patients who are eligible to receive full treatment regimen, but the use of evaluation scales should not bring the risk of under-treating patients. Thus, a multidisciplinary approach involving geriatricians and haemato-oncologists is necessary.

CGA could be a simple, feasible, and cost-effective screening tool to identify patients who are more at risk of developing cardiac dysfunction in order to establish the need for further investigation and/or for a more stringent cardio-oncology follow-up. Further investigation is necessary to clarify how cancer, cardiovascular dysfunction, and frailty interact.

## Figures and Tables

**Table 1:** Clinical and oncological characteristics of the population.

Variables	All (*n* = 108)	Group 1 (*n* = 54)	Group 2 (*n* = 34)	Group 3 (*n* = 20)	*p*-Value
***Age***	73.55 ± 5.43	73.32 ± 5.40	73.46 ± 5.09	74.34 ± 6.23	1
***Female, n (%)***	44 (40.7)	18 (33.3)	15 (44.1)	11 (55)	0.211
***Comorbidities***					
Diabetes mellitus, ***n*** (%)	30 (27.7)	16 (29.6)	8 (23.5)	6 (30)	0.844
Hypertension, ***n*** (%)	83 (76.8)	44 (81.4)	25 (73.5)	14 (70)	0.466
Dyslipidemia, ***n*** (%)	49 (45.3)	25 (46.2)	16 (47)	8 (40)	0.903
Dysthyroidism, ***n*** (%)	9 (8.3)	4 (7.4)	3 (8.8)	2 (10)	0.903
Active smoking, ***n*** (%)	16 (14.8)	7 (12.9)	7 (20.5)	2 (10)	0.585
Previous smoking, ***n*** (%)	37 (34.2)	23 (42.5)	8 (23.5)	6 (30)	0.188
LV dysfunction, ***n*** (%)	24 (22.2)	8 (14.8)	12 (35.2)	4 (20)	0.087
Atrial fibrillation, ***n*** (%)	17 (15.7)	9 (16.6)	5 (14.7)	3 (15)	1
COPD, ***n*** (%)	15 (13.8)	11 (20.3)	2 (5.8)	2 (10)	0.164
Carotid atherosclerosis, ***n*** (%)	29 (26.8)	16 (29.6)	8 (23,5)	5 (25)	0.843
BMI 25–29.9 overweight, ***n*** (%)	47 (43.5)	27 (50)	11 (32.3)	9 (45)	0.286
BMI ≥ 30, obese, ***n*** (%)	14 (12.9)	7 (12.9)	4 (11.7)	3 (15)	1
Heart failure, ***n*** (%)	24 (22)	8 (15)	12 (35)	4 (20)	0.077
Previous MI, ***n*** (%)	20 (19)	13 (24)	5 (15)	2 (10)	0.302
Arrhythmias, ***n*** (%)	23 (21)	12 (22)	7 (21)	3 (15)	0.790
***Cancer type***					
Colon, ***n*** (%)	42 (38.9)	25 (46.3)	14 (41.2)	3 (15)	**0.038**
Gastric, ***n*** (%)	7 (6.5)	3 (5.6)	1 (2.9)	3 (15)	0.242
Genital, ***n*** (%)	1 (0.9)	1 (1.9)	0 (0)	0 (0)	1
Larynx, ***n*** (%)	3 (2.8)	2 (3.7)	1 (2.9)	0 (0)	1
Hodgkin’s lymphoma, ***n*** (%)	4 (3.7)	3 (5.6)	0 (0)	1 (5)	0.398
Non-Hodgkin’s lymphoma, ***n*** (%)	10 (9.3)	7 (13.0)	0 (0)	3 (15)	**0.049**
Tongue, ***n*** (%)	2 (1.9)	1 (1.9)	1 (2.9)	0 (0)	1
Breast, ***n*** (%)	13 (12.0)	2 (3.7)	6 (17.6)	5 (25)	0.015
Melanoma, ***n*** (%)	4 (3.7)	3 (5.6)	1 (2.9)	0 (0)	0.818
Ovarian, ***n*** (%)	1 (0.9)	0 (0)	1 (2.9)	0 (0)	0.5
Pancreas, ***n*** (%)	3 (2.8)	0 (0)	2 (5.9)	1 (5)	0.172
Parotid, ***n*** (%)	1 (0.9)	1 (1.9)	0 (0)	0 (0)	1
Pleura, ***n*** (%)	1 (0.9)	0 (0)	1 (2.9)	0 (0)	0.5
Polycythemia vera, ***n*** (%)	1 (0.9)	0 (0)	1 (2.9)	0 (0)	0.5
Lung, ***n*** (%)	7 (6.5)	2 (3.7)	3 (8.8)	2 (10)	0.390
Prostate, ***n*** (%)	4 (3.7)	2 (3.7)	1 (2.9)	1 (5)	1
Kidney, ***n*** (%)	1 (0.9)	0 (0)	0 (0)	1 (5)	0.185
Sarcoma, ***n*** (%)	1 (0.9)	1 (1.9)	0 (0)	0 (0)	1
Thyroid, ***n*** (%)	1 (0.9)	1 (1.9)	0 (0)	0 (0)	1
Biliary tract, ***n*** (%)	1 (0.9)	0 (0)	1 (2.9)	0 (0)	0.5
***Anticancer drugs (ongoing or previously taken), n (%)***					
VEGF-based and/or EGFR-based protocols (± PA ± PDC ± other chemotherapeutic agents)	29 (26.8)	12 (22.2)	16 (47)	1 (5)	0.002
PA-based and/or PDC-based protocols (± other chemotherapeutic agents)	33 (30.5)	21 (38.8)	8 (23.5)	4 (20)	0.184
Anthracyclines-based protocols	9 (8.3)	2 (3.7)	5 (14.7)	2 (10)	0.160
Non-anthracyclines-based schemes for lymphomas	8 (7.4)	7 (12.9)	0	1 (5)	0.056
MEKi ± BRAFi	1 (0.9)	0	1 (2.9)	0	0.500
Immunotherapy	7 (6.4)	5 (9.2)	0	2 (10)	0.144
Hormone-based protocols	3 (2.7)	0	0	3 (15)	0.006
Others	18 (16.6)	7 (12.9)	4 (11.7)	7 (35)	0.072

Discrete variables are expressed as absolute number (percentage). Continuous variables are expressed as the mean ± standard deviation, as they are normally distributed. List of abbreviations: BRAFi, BRAF inhibitors; BMI, body mass index; COPD, chronic obstructive pulmonary disease; EGFR, endothelia growth factor receptor; LV, left ventricular; MEKi, Mitogen-activated protein kinase inhibitors; MI, myocardial infarction; PA, purine analogues; PDC, platinum-derived compounds; VEGF, vascular endothelial growth factor.

**Table 2:** Clinical outcomes according to the three groups.

	All (*n* = 108)	Group 1 (*n* = 54)	Group 2 (*n* = 34)	Group 3 (*n* = 20)	*p*-Value
CVEs during follow-up, ***n*** (%)	32 (29.6)	8 (14.8)	21 (61.8)	3 (15)	**<0.001**
Cancer treatment modification or temporary suspension, ***n*** (%)	12 (11.1)	2 (3.7)	10 (29.4)	NA	**<0.001**
Cancer treatment withdrawal, ***n*** (%)	3 (2.8)	0 (0)	3 (8.8)	NA	**0.035**
Death for all causes, ***n*** (%)	32 (29.6)	12 (22.2)	16 (47.1)	4 (20)	**0.034**

List of abbreviations: CVE, cardiovascular events.

**Table 3:** Modifications of cardiovascular therapies in the study cohort during follow-up.

	All (*n* = 108)	Group 1 (*n* = 54)	Group 2 (*n* = 34)	Group 3 (*n* = 20)	*p*-Value
***Cardiovascular treatment optimization***					
Beta-blockers, ***n*** (%)	19 (17.6)	9 (16.7)	7 (20.6)	3 (15)	0.892
ACE-inhibitors, ***n*** (%)	8 (7.4)	4 (7.4)	2 (5.9)	2 (10)	0.792
ARBs, ***n*** (%)	6 (5.6)	2 (3.7)	2 (5.9)	2 (10)	0.792
ARNIs, ***n*** (%)	1 (0.9)	0 (0)	1 (2.9)	0 (0)	0.5
Diuretics, ***n*** (%)	6 (5.6)	5 (9.3)	0 (0)	1 (5)	0.228
Calcium channel blockers, ***n*** (%)	5 (4.6)	4 (7.4)	1 (2.9)	0 (0)	0.580
Statin, ***n*** (%)	13 (12)	9 (16.7)	3 (8.8)	1 (5)	0.356
Antiplatelet, ***n*** (%)	3 (2.8)	1 (1.9)	2 (5.9)	0 (0)	0.582
***Cardiovascular treatment initiation***					
Beta-blockers, ***n*** (%)	31 (28.7)	15 (27.8)	11 (32.4)	5 (25)	0.883
ACE-inhibitors, ***n*** (%)	7 (6.5)	2 (3.7)	2 (5.9)	3 (15)	0.520
ARBs, ***n*** (%)	5 (4.6)	3 (5.6)	2 (5.9)	0 (0)	0.705
ARNIs, ***n*** (%)	2 (1.9)	0 (0)	2 (5.9)	0 (0)	0.130
Diuretics, ***n*** (%)	22 (20.4)	9 (16.7)	7 (20.6)	6 (30)	0.436
Calcium channel blockers, ***n*** (%)	8 (7.4)	5 (9.3)	0 (0)	3 (15)	0.067
Statin, ***n*** (%)	27 (25)	15 (27.8)	8 (23.5)	4 (20)	0.837
Antiplatelet, ***n*** (%)	33 (30.6)	16 (29.6)	13 (38.2)	4 (20)	0.396
Anticoagulation, ***n*** (%)	12 (11.1)	7 (13)	3 (8.8)	2 (10)	0.918

List of abbreviations: ACE, angiotensin-converting enzyme; ARBs, angiotensin II receptor blockers; ARNIs, angiotensin receptor-neprilysin inhibitors.

**Table 4:** Differences in terms of characteristics between patients with the CGA and those without.

	With CGA (*n* = 62)	No CGA (*n* = 46)	*p*-Value
Age, years	73 ± 5	74 ± 6	0.809
Female, ***n*** (%)	24 (39)	20 (43)	0.844
SBP, mmHg	135 ± 19	134 ± 16	0.847
DBP, mmHg	80 ± 11	81 ± 10	0.725
HR, bpm	72 ± 17	72 ± 18	0.881
LVEF, %	53 ± 8	52 ± 9	0.648
Hypertension, ***n*** (%)	47 (76)	36 (78)	0.765
Diabetes, ***n*** (%)	20 (33)	10 (21)	0.202
Obese, ***n*** (%)	7 (12)	7 (15)	0.774
Death, ***n*** (%)	19 (31)	13 (28)	0.832
Dyslipidemia, ***n*** (%)	30 (49)	19 (40)	0.437
HF, ***n*** (%)	13 (21)	11 (23)	0.819
Group 1, ***n*** (%)	30 (49)	24 (51)	0.352
Group 2, ***n*** (%)	17 (28)	17 (36)	
Group 3, ***n*** (%)	14 (23)	6 (13)	
Beta blockers, ***n*** (%)	31 (51)	23 (49)	1.000
ARBs, ***n*** (%)	14 (23)	19 (40)	0.060
ACE-I, ***n*** (%)	28 (46)	15 (32)	0.168
Statins, ***n*** (%)	22 (38)	15 (33)	0.681
diuretics, ***n*** (%)	18 (30)	19 (40)	0.307
CCBs, ***n*** (%)	17 (28)	10 (21)	0.505

List of abbreviations: ACE-I, angiotensin-converting enzyme inhibitors; ARBs, angiotensin receptor blockers; CCBs, calcium channel blockers; CGA, comprehensive geriatric assessment; DBP, diastolic blood pressure; HF, heart failure; HR, heart rate; LVEF, left ventricular ejection fraction; SBP, systolic blood pressure.

**Table 5:** Comprehensive geriatric assessment (CGA) according to the groups.

Variables	All (*n* = 62)	Group 1 (*n* = 30)	Group 2 (*n* = 18)	Group 3 (*n* = 14)	*p*-Value
Age, yrs	73.55 ± 5.41	73.63 ± 4.86	73.69 ± 5.41	73.19 ± 6.81	0.962
Females, ***n*** (%)	25 (40)	11 (37)	8 (44)	6 (43)	0.847
NYHA-FC III-V, ***n*** (%)	2 (3.2)	1 (3.3)	0	1 (7.1)	0.618
MMSE	26.4 ± 3.4	26.4 ± 3.6	25.8 ± 2.7	27.3 ± 3.4	0.445
GDS	3.9 ± 3.6	3.4 ± 3.2	5.2 ± 4.4	3.4 ± 2.9	0.219
BADL	0.7 ± 1.1	0.5 ± 0.9	0.9 ± 1.2	0.7 ± 1.5	0.554
IADL	1.9 ± 2.4	1.4 ± 2.1	2.7 ± 2.8	2.2 ± 2.5	0.181
TINETTI	24.5 ± 4.1	25.2 ± 3.4	23 ± 5	25 ± 3.7	0.164
SPPB	7 ± 3.4	7.6 ± 3.2	6.2 ± 3.7	6.7 ± 3.5	0.361
MNA	23.5 ± 3.7	23.43 ± 3.8	23.3 ± 3.9	24.1 ± 3.5	0.814
CIRS-C	4.5 ± 1.9	5 ± 1.9	4.1 ± 1.8	4.2 ± 2	0.234
CIRS-G	2.1 ± 0.7	2.2 ± 1	2 ± 0.4	1.8 ± 0.3	0.278
Drug number, n	6.7 ± 3.3	6.7 ± 3	7.1 ± 3.1	6.5 ± 4.4	0.876
PASE	78.6 ± 64.7	89.9 ± 65.8	46.2 ± 55.3	93.3 ± 63.1	0.060
SSA	5.6 ± 2.6	5.2 ± 2.3	6.4 ± 3	5.5 ± 2.5	0.292
IFI	12.2 ± 7	10.7 ± 5.75	14.1 ± 7.9	12.7 ± 8	0.255

List of abbreviations: BADL, Basic Activity of Daily Living; CIRS-C, Cumulative Illness Rating Scale—Comorbidities; CIRS-G, Cumulative Illness Rating Scale-Geriatric; GDS, Geriatric Depression Scale; IADL, Instrumental Activity of Daily Living; IFI, Italian Frailty Index; MMSE, Mini-Mental State Examination; MNA, Mini Nutritional Assessment; NYHA-FC, New York Heart Association-functional class; PASE, Physical Activity Scale for the Elderly; SSA, Social Support Assessment; SPPB, Short Performance Physical Battery; TINETTI, Tinetti Scale for balance and gait evaluation.

**Table 6:** Echocardiographic parameters according to groups.

	All (*n* = 62)	Group 1 (*n* = 30)	Group 2 (*n* = 18)	Group 3 (*n* = 14)	*p*-Value
LV-IDd, mm	49 ± 5.7	48.5 ± 5.1	49.6 ± 7	50.2 ± 5.2	0.615
LV-STd, mm	11 ± 1.5	10.7 ± 1.4	10.7 ± 1.4	10.9 ± 1.7	0.94
LV-PWTd, mm	9 ± 1.1	9.6 ± 1.1	9 ± 1.3	9.3 ± 1	0.313
RWT	0 ± 0.05	0.3 ± 0.05	0.3 ± 0.06	0.3 ± 0.05	0.339
LV mass i, g/m^2^	103 ± 23.2	102.8 ± 21.2	100 ± 27.4	104.1 ± 24.4	0.884
LV-EDVi, mL/m^2^	56 ± 14.7	53.2 ± 12.5	56.7 ± 17.1	60.8 ± 15.3	0.275
LV-EF, %	53 ± 7.7	54.7 ± 5.5	49.7 ± 9.3	51.8 ± 8.4	0.079
E wave, cm/s	65 ± 21.9	71.1 ± 22.8	61.1 ± 19.22	56.9 ± 20.9	0.089
A wave, cm/s	83 ± 26	83.8 ± 24.2	81.7 ± 30.8	82.8 ± 24.3	0.964
Dec Time, ms	241 ± 72.1	244.9 ± 73.6	225.2 ± 78.5	247.3 ± 66	0.615
E/A	1 ± 0.3	0.8 ± 0.3	0.8 ± 0.3	0.6 ± 0.2	0.167
E/e’	19 ± 4.2	9.4 ± 3	10.9 ± 5.1	10.6 ± 4.8	0.447
LAVi, mL/m^2^	37 ± 10.8	38 ± 11.5	34.6 ± 8	39.1 ± 12.5	0.458
TAPSE, mm	21 ± 3.4	21.2 ± 3.4	21.9 ± 4.2	20.3 ± 2	0.443
S’, cm/s	12 ± 2.7	12.1 ± 2.6	12.8 ± 3.5	12.7 ± 1.8	0.652
PASP, mmHg	32 ± 10.1	31.4 ± 9.6	30.5 ± 10	34.1 ± 11.7	0.598

List of abbreviations: Dec time, deceleration time; IDd, internal diameter during diastole; LAVi, left atrial volume indexed; LV-EDVi, left ventricular-end diastolic volume indexed; LV, left ventricle; LV-EF, left ventricular ejection fraction; PASP, pulmonary arterial systolic pressure; PWTd, posterior wall thickness during diastole; RWT, relative wall thickness; STd, septum thickness during diastole; TAPSE, tricuspid annular plane systolic excursion.

**Table 7:** Comprehensive geriatric assessment (CGA) between survived patients (subgroup A) and patients who died (subgroup B).

Variables	Subgroup A (*n* = 43)	Subgroup B (*n* = 19)	*p*-Value
MMSE	26.1 ± 3.6	27.2 ± 2.6	0.263
GDS	3.6 ± 3.6	4.7 ± 3.5	0.268
BADL	0.4 ± 0.7	1.2 ± 1.7	0.074
IADL	1.6 ± 2.3	2.8 ± 2.7	0.071
TINETTI	25.1 ± 3.6	23.2 ± 4.8	0.103
SPPB	7.5 ± 3.2	4.7 ± 3.6	0.060
MNA	24.1 ± 3.3	22.2 ± 4.3	0.073
CIRS-C	4.4 ± 2	4.8 ± 1.7	0.379
CIRS-G	2.1 ± 0.9	2.1 ± 0.4	0.988
Drugs number, n	6 ± 2.6	8.4 ± 4.2	**0.035**
PASE	93.8 ± 62.3	41.8 ± 53.3	**0.004**
SSA	5.5 ± 2.7	5.9 ± 2.4	0.555
IFI	11.6 ± 6.6	13.5 ± 7.8	0.338

List of abbreviations: BADL, Basic Activity of Daily Living; CIRS-C, Cumulative Illness Rating Scale—Comorbidities; CIRS-G, Cumulative Illness Rating Scale-Geriatric; GDS, Geriatric Depression Scale; IADL, Instrumental Activity of Daily Living; IFI, Italian Frailty Index; MMSE, Mini-Mental State Examination; MNA, Mini Nutritional Assessment; PASE, Physical Activity Scale for the Elderly; SSA, Social Support Assessment; SPPB, Short Performance Physical Battery; TINETTI, Tinetti Scale for balance and gait evaluation.

**Table 8:** Echocardiographic measurement comparison between survived patients (subgroup A) and patients who died (subgroup B).

	Subgroup A (*n* = 43)	Subgroup B (*n* = 19)	*p*-Value
LV-IDd, mm	48.8 ± 5.6	50.1 ± 5.9	0.435
LV-STd, mm	10.4 ± 1.4	11.5 ± 1.4	**0.009**
LV-PWTd, mm	9.3 ± 1.2	9.5 ± 1.1	0.404
RWT	0.3 ± 0.06	0.3 ± 0.04	0.627
LV mass i, g/m^2^	97.2 ± 19.1	113.2 ± 28.5	**0.036**
LV-EDVi, mL/m^2^	56.6 ± 15.6	54.4 ± 12.6	0.578
LV-EF, %	52.5 ± 8.2	52.8 ± 6.4	0.895
E wave, cm/sec	67.4 ± 22.3	59.5 ± 20.5	0.197
A wave, cm/sec	80.6 ± 21.8	87.6 ± 33.2	0.341
Dec Time, ms	232.9 ± 78.1	254 ± 59.6	0.302
E/A	0.8 ± 0.3	0.7 ± 0.1	0.099
E/e’	10.1 ± 4.4	10.1 ± 3.6	0.973
LAVi, mL/m^2^	38.5 ± 11.8	34.5 ± 7.8	0.192
TAPSE, mm	21.1 ± 3.4	21.4 ± 3.6	0.808
S’, cm/sec	12 ± 2.7	13.3 ± 2.6	0.111
PASP, mmHg	31.3 ± 9.4	32.7 ± 11.9	0.625

List of abbreviations: Dec time, deceleration time; IDd, internal diameter during diastole; LAVi, left atrial volume indexed; LV-EDVi, left ventricular-end diastolic volume indexed; LV, left ventricle; LV-EF, left ventricular ejection fraction; PASP, pulmonary arterial systolic pressure; PWTd, posterior wall thickness during diastole; RWT, relative wall thickness; STd, septum thickness during diastole; TAPSE, tricuspid annular plane systolic excursion.

## Data Availability

Data are available upon reasonable request to the authors.

## Abbreviations

The following abbreviations are used in this manuscript:

BADL	Basic Activity of Daily Living; IADL
BMI	body mass index
CIRS-C	Cumulative Illness Rating Scale Cumulative—Comorbidities
CIRS-G	Cumulative Illness Rating Scale Cumulative—Geriatric
COPD	chronic obstructive pulmonary disease
Dec Time	deceleration time
GDS	Geriatric Depression Scale
IADL	Instrumental Activity of Daily Living
IDd	internal diameter during diastole
IFI	Italian fragility index
LAVi	left atrial volume indexed
LV	Left ventricle
LV-EDVi	left ventricular-end diastolic volume indexed
LV-EF	left ventricular ejection fraction
MMSE	Mini-Mental State Examination
MNA	Mini Nutritional Assessment
NYHA-FC	New York Association-Functional Class
PASE	Physical Activity Scale for the Elderly
PASP	pulmonary arterial systolic pressure
PWTd	posterior wall thickness during diastole
RWT	relative wall thickness
SPPB	Short Performance Physical Battery
SSA	Social Support Assessment
STd	septum thickness during diastole
TAPSE	Tricuspid annular plane systolic excursion
TINETTI	Tinetti Scale for balance and gait evaluation

## References

[1] Anker M.S., Von Haehling S., Landmesser U., Coats A.J.S., Anker S.D. (2018). Cancer and heart failure-more than meets the eye: Common risk factors and co-morbidities. Eur. J. Heart Fail..

[2] Liguori I., Russo G., Curcio F., Bulli G., Aran L., Della-Morte D., Gargiulo G., Testa G., Cacciatore F., Bonaduce D. (2018). Oxidative stress, aging, and diseases. Clin. Interv. Aging.

[3] De Boer R.A., Meijers W.C., Van Der Meer P., Van Veldhuisen D.J. (2019). Cancer and heart disease: Associations and relations. Eur. J. Heart Fail..

[4] Libby P., Kobold S. (2019). Inflammation: A common contributor to cancer, aging, and cardiovascular diseases-expanding the concept of cardio-oncology. Cardiovasc. Res..

[5] Meijers W.C., De Boer R.A. (2019). Common risk factors for heart failure and cancer. Cardiovasc. Res..

[6] Scotte F., Bossi P., Carola E., Cudennec T., Dielenseger P., Gomes F., Knox S., Strasser F. (2018). Addressing the quality of life needs of older patients with cancer: A SIOG consensus paper and practical guide. Ann. Oncol..

[7] Russo C., Giannotti C., Signori A., Cea M., Murialdo R., Ballestrero A., Scabini S., Romairone E., Odetti P., Nencioni A. (2018). Predictive values of two frailty screening tools in older patients with solid cancer: A comparison of SAOP2 and G8. Oncotarget.

[8] Abete P., Basile C., Bulli G., Curcio F., Liguori I., Della-Morte D., Gargiulo G., Langellotto A., Testa G., Galizia G. (2017). The Italian version of the “frailty index” based on deficits in health: A validation study. Aging Clin. Exp. Res..

[9] Clegg A., Young J., Iliffe S., Rickett M.O., Rockwood K. (2014). Frailty in Elderly. Lancet.

[10] Handforth C., Clegg A., Young A., Simpkins S., Seymour M.T., Selby P.J., Young J. (2014). The prevalence and outcomes of frailty in older cancer patients: A systematic review. Ann. Oncol..

[11] Denfeld Q.E., Winters-Stone K., Mudd J.O., Gelow J.M., Kurdi S., Lee C.S. (2017). The prevalence of frailty in heart failure: A systematic review and meta-analysis. Int. J. Cardiol..

[12] Shen L., Jhund P.S., Petrie M.C., Claggett B.L., Barlera S., Cleland J.G.F., Dargie H.J., Granger C.B., Kjekshus J., Kober L. (2017). Declining Risk of Sudden Death in Heart Failure. N. Engl. J. Med..

[13] Conrad N., Judge A., Canoy D., Tran J., Pinho-Gomes A.C., Millett E.R.C., Salimi-Khorshidi G., Cleland J.G., Mcmurray J.J.V., Rahimi K. (2019). Temporal Trends and Patterns in Mortality After Incident Heart Failure: A Longitudinal Analysis of 86000 Individuals. JAMA Cardiol..

[14] Moliner P., Lupon J., De Antonio M., Domingo M., Santiago-Vacas E., Zamora E., Cediel G., Santesmases J., Diez-Quevedo C., Troya M.I. (2019). Trends in modes of death in heart failure over the last two decades: Less sudden death but cancer deaths on the rise. Eur. J. Heart Fail..

[15] Perez I.E., Taveras Alam S., Hernandez G.A., Sancassani R. (2019). Cancer Therapy-Related Cardiac Dysfunction: An Overview for the Clinician. Clin. Med. Insights Cardiol..

[16] Bertero E., Ameri P., Maack C. (2019). Bidirectional Relationship Between Cancer and Heart Failure: Old and New Issues in Cardio-oncology. Card. Fail. Rev..

[17] Parker S.G., McCue P., Phelps K., McCleod A., Arora S., Nockels K., Kennedy S., Roberts H., Conroy S. (2018). What is Comprehensive Geriatric Assessment (CGA)? An umbrella review. Age Ageing.

[18] Todaro M.C., Oreto L., Qamar R., Paterick T.E., Carerj S., Khandheria B.K. (2013). Cardioncology: State of the heart. Int. J. Cardiol..

[19] Cuomo A., Mercurio V., Varricchi G., Galdiero M.R., Rossi F.W., Carannante A., Arpino G., Formisano L., Bianco R., Carlomagno C. (2022). Impact of a cardio-oncology unit on prevention of cardiovascular events in cancer patients. ESC Heart Fail..

[20] Lyon A.R., López-Fernández T., Couch L.S., Asteggiano R., Aznar M.C., Bergler-Klein J., Boriani G., Cardinale D., Cordoba R., Cosyns B. (2022). 2022 ESC Guidelines on cardio-oncology developed in collaboration with the European Hematology Association (EHA), the European Society for Therapeutic Radiology and Oncology (ESTRO) and the International Cardio-Oncology Society (IC-OS). Eur. Heart J..

[21] Čelutkienė J., Pudil R., López-Fernández T., Grapsa J., Nihoyannopoulos P., Bergler-Klein J., Cohen-Solal A., Farmakis D., Tocchetti C.G., von Haehling S. (2020). Role of cardiovascular imaging in cancer patients receiving cardiotoxic therapies: A position statement on behalf of the Heart Failure Association (HFA), the European Association of Cardiovascular Imaging (EACVI) and the Cardio-Oncology Council of the European Society of Cardiology (ESC). Eur. J. Heart Fail..

[22] Lyon A.R., Dent S., Stanway S., Earl H., Brezden-Masley C., Cohen-Solal A., Tocchetti C.G., Moslehi J.J., Groarke J.D., Bergler-Klein J. (2020). Baseline cardiovascular risk assessment in cancer patients scheduled to receive cardiotoxic cancer therapies: A position statement and new risk assessment tools from the Cardio-Oncology Study Group of the Heart Failure Association of the European Society of Cardiology in collaboration with the International Cardio-Oncology Society. Eur. J. Heart Fail..

[23] Pudil R., Mueller C., Čelutkienė J., Henriksen P.A., Lenihan D., Dent S., Barac A., Stanway S., Moslehi J., Suter T.M. (2020). Role of serum biomarkers in cancer patients receiving cardiotoxic cancer therapies: A position statement from the Cardio-Oncology Study Group of the Heart Failure Association and the Cardio-Oncology Council of the European Society of Cardiology. Eur. J. Heart Fail..

[24] Lang R.M., Badano L.P., Mor-Avi V., Afilalo J., Armstrong A., Ernande L., Flachskampf F.A., Foster E., Goldstein S.A., Kuznetsova T. (2015). Recommendations for cardiac chamber quantification by echocardiography in adults: An update from the American Society of Echocardiography and the European Association of Cardiovascular Imaging. Eur. Heart J. Cardiovasc. Imaging.

[25] Nagueh S.F., Smiseth O.A., Appleton C.P., Byrd B.F., Dokainish H., Edvardsen T., Flachskampf F.A., Gillebert T.C., Klein A.L., Lancellotti P. (2016). Recommendations for the evaluation of left ventricular diastolic function by echocardiography: An update from the American Society of Echocardiography and the European Association of Cardiovascular Imaging. J. Am. Soc. Echocardiogr..

[26] Ewer M.S., Lenihan D.J. (2008). Left ventricular ejection fraction and cardiotoxicity: Is our ear really to the ground?. J. Clin. Oncol..

[27] Folstein M.F., Folstein S.E., McHugh P.R. (1975). “Mini-mental state”. A practical method for grading the cognitive state of patients for the clinician. J. Psychiatr. Res..

[28] Yesavage J.A., Brink T.L., Rose T.L., Lum O., Huang V., Adey M., Leirer V.O. (1982). Development and validation of a geriatric depression screening scale: A preliminary report. J. Psychiatr. Res..

[29] Linn B.S., Linn M.W., Gurel L. (1968). Cumulative illness rating scale. J. Am. Geriatr. Soc..

[30] Katz S., Ford A.B., Moskowitz R.W., Jackson B.A., Jaffe M.W. (1963). Studies of illness in the aged. The index of ADL: A standardized measure of biological and psychological function. JAMA.

[31] Lawton M.P., Brody E.M. (1969). Assessment of older people: Self-maintaining and instrumental activities of daily living. Gerontologist.

[32] Kaiser M.J., Bauer J.M., Rämsch C., Uter W., Guigoz Y., Cederholm T., Thomas D.R., Anthony P.S., Charlton K.E., Maggio M. (2010). Frequency of malnutrition in older adults: A multinational perspective using the mini nutritional assessment. J. Am. Geriatr. Soc..

[33] Tinetti M.E., Richman D., Powell L. (1990). Falls efficacy as a measure of fear of falling. J. Gerontol..

[34] Guralnik J.M., Simonsick E.M., Ferrucci L., Glynn R.J., Berkman L.F., Blazer D.G., Scherr P.A., Wallace R.B. (1994). A short physical performance battery assessing lower extremity function: Association with self-reported disability and prediction of mortality and nursing home admission. J. Gerontol..

[35] Washburn R.A., Smith K.W., Jette A.M., Janney C.A. (1993). The Physical Activity Scale for the Elderly (PASE): Development and evaluation. J. Clin. Epidemiol..

[36] Mazzella F., Cacciatore F., Galizia G., Della-Morte D., Rossetti M., Abbruzzese R., Langellotto A., Avolio D., Gargiulo G., Ferrara N. (2010). Social support and long-term mortality in the elderly: Role of comorbidity. Arch. Gerontol. Geriatr..

[37] Searle S.D., Mitnitski A., Gahbauer E.A., Gill T.M., Rockwood K. (2008). A standard procedure for creating a frailty index. BMC Geriatr..

[38] de Boer R.A., Hulot J.S., Tocchetti C.G., Aboumsallem J.P., Ameri P., Anker S.D., Bauersachs J., Bertero E., Coats A.J., Čelutkienė J. (2020). Common mechanistic pathways in cancer and heart failure. A scientific roadmap on behalf of the Translational Research Committee of the Heart Failure Association (HFA) of the European Society of Cardiology (ESC). Eur. J. Heart Fail..

[39] Cuomo A., Pirozzi F., Attanasio U., Franco R., Elia F., De Rosa E., Russo M., Ghigo A., Ameri P., Tocchetti C.G. (2020). Cancer risk in the heart failure population: Epidemiology, mechanisms, and clinical implications. Curr. Oncol. Rep..

[40] Mercurio V., Cuomo A., Cadeddu Dessalvi C., Deidda M., Di Lisi D., Novo G., Manganaro R., Zito C., Santoro C., Ameri P. (2020). Redox imbalances in ageing and metabolic alterations: Implications in cancer and cardiac diseases. An overview from the Working Group of Cardiotoxicity and Cardioprotection of the Italian Society of Cardiology (SIC). Antioxidants.

[41] Narayan H.K., Finkelman B., French B., Plappert T., Hyman D., Smith A.M., Margulies K.B., Ky B. (2017). Detailed echocardiographic phenotyping in breast cancer patients: Associations with ejection fraction decline, recovery, and heart failure symptoms over 3 years of follow-up. Circulation.

[42] Moslehi J., Zhang Q., Moore K.J. (2020). Crosstalk between the heart and cancer. Circulation.

[43] Lancellotti P., Suter T.M., López-Fernández T., Galderisi M., Lyon A.R., Van der Meer P., Cohen Solal A., Zamorano J.L., Jerusalem G., Moonen M. (2018). Cardio-Oncology Services: Rationale, organization, and implementation. Eur. Heart J..

[44] Zamorano J.L., Gottfridsson C., Asteggiano R., Atar D., Badimon L., Bax J.J., Cardinale D., Cardone A., Feijen E.A.M., Ferdinandy P. (2020). The cancer patient and cardiology. Eur. J. Heart Fail..

[45] Pareek N., Cevallos J., Moliner P., Shah M., Tan L.L., Chambers V., Baksi A.J., Khattar R.S., Sharma R., Rosen S.D. (2018). Activity and outcomes of a cardio-oncology service in the United Kingdom—A five-year experience. Eur. J. Heart Fail..

[46] Francis S.A., Asnani A., Neilan T., Scherrer-Crosbie M. (2015). Optimizing cardio-oncology programs for cancer patients. Future Oncol..

[47] Jung H., Kim S., Lee C.S., Byeon S.H., Kim S.S., Lee S.W., Kim Y.J. (2024). Real-World Incidence of Incident Noninfectious Uveitis in Patients Treated with BRAF Inhibitors: A Nationwide Clinical Cohort Study. Am. J. Ophthalmol..

[48] Landi F., Liperoti R., Russo A., Capoluongo E., Barillaro C., Pahor M., Bernabei R., Onder G. (2010). Disability, more than multimorbidity, was predictive of mortality among older persons aged 80 years and older. J. Clin. Epidemiol..

[49] Studenski S., Perera S., Patel K., Rosano C., Faulkner K., Inzitari M., Brach J., Chandler J., Cawthon P., Connor E.B. (2011). Gait Speed and Survival in Older Adults. JAMA.

[50] Chiarantini D., Volpato S., Sioulis F., Bartalucci F., Del Bianco L., Mangani I., Pepe G., Tarantini F., Berni A., Marchionni N. (2010). Lower extremity performance measures predict long-term prognosis in older patients hospitalized for heart failure. J. Card. Fail..

[51] Caccialanza R., Pedrazzoli P., Cereda E., Gavazzi C., Pinto C., Paccagnella A., Beretta G.D., Nardi M., Laviano A., Zagonel V. (2016). Nutritional Support in Cancer Patients: A Position Paper from the Italian Society of Medical Oncology (AIOM) and the Italian Society of Artificial Nutrition and Metabolism (SINPE). J. Cancer.

[52] Nightingale G., Skonecki E., Boparai M.K. (2017). The Impact of Polypharmacy on Patient Outcomes in Older Adults with Cancer. Cancer J..

[53] Arends J., Bachmann P., Baracos V., Barthelemy N., Bertz H., Bozzetti F., Fearon K., Hütterer E., Isenring E., Kaasa S. (2017). ESPEN guidelines on nutrition in cancer patients. Clin. Nutr..

[54] van Nieuwenhuizen A.J., Buffart L.M., van Uden-Kraan C.F., van der Velden L.A., Lacko M., Brug J., Leemans C.R., Verdonck-de Leeuw I.M. (2018). Patient-reported physical activity and the association with health-related quality of life in head and neck cancer survivors. Support. Care Cancer.

[55] Holmes M.D., Chen W.Y., Feskanich D., Kroenke C.H., Colditz G.A. (2005). Physical activity and survival after breast cancer diagnosis. JAMA.

[56] Kenfield S.A., Stampfer M.J., Giovannucci E., Chan J.M. (2011). Physical activity and survival after prostate cancer diagnosis in the health professionals follow-up study. J. Clin. Oncol..

[57] Pardal E., Díez Baeza E., Salas Q., García T., Sancho J.M., Monzón E., Moraleda J.M., Córdoba R., de la Cruz F., Queizán J.A. (2018). A new prognostic model identifies patients aged 80 years and older with diffuse large B-cell lymphoma who may benefit from curative treatment: A multicenter, retrospective analysis by the Spanish GELTAMO group. Am. J. Hematol..

[58] Nabhan C., Smith S.M., Helenowski I., Ramsdale E., Parsons B., Karmali R., Feliciano J., Hanson B., Smith S., McKoy J. (2012). Analysis of very elderly (≥80 years) non-hodgkin lymphoma: Impact of functional status and co-morbidities on outcome. Br. J. Haematol..

[59] Wieringa A., Boslooper K., Hoogendoorn M., Joosten P., Beerden T., Storm H., Kibbelaar R.E., Veldhuis G.J., van Kamp H., van Rees B. (2014). Comorbidity is an independent prognostic factor in patients with advanced-stage diffuse large B-cell lymphoma treated with R-CHOP: A population-based cohort study. Br. J. Haematol..

[60] Saygin C., Jia X., Hill B., Dean R., Pohlman B., Smith M.R., Jagadeesh D. (2017). Impact of comorbidities on outcomes of elderly patients with diffuse large B-cell lymphoma. Am. J. Hematol..

[61] Tucci A., Martelli M., Rigacci L., Riccomagno P., Cabras M.G., Salvi F., Stelitano C., Fabbri A., Storti S., Fogazzi S. (2015). Comprehensive geriatric assessment is an essential tool to support treatment decisions in elderly patients with diffuse large B-cell lymphoma: A prospective multicenter evaluation in 173 patients by the Lymphoma Italian Foundation (FIL). Leuk. Lymphoma.

[62] Nadruz Jr W., Kitzman D., Windham B.G., Kucharska-Newton A., Butler K., Palta P., Griswold M.E., Wagenknecht L.E., Heiss G., Solomon S.D. (2017). Cardiovascular Dysfunction and Frailty Among Older Adults in the Community: The ARIC Study. J. Gerontol. A Biol. Sci. Med. Sci..

[63] Kusunose K., Okushi Y., Yamada H., Nishio S., Torii Y., Hirata Y., Saijo Y., Ise T., Yamaguchi K., Yagi S. (2018). Prognostic Value of Frailty and Diastolic Dysfunction in Elderly Patients. Circ. J..

[64] Abdar Esfahani M., Mokarian F., Karimipanah M. (2017). Alterations in the echocardiographic variables of the right ventricle in asymptomatic patients with breast cancer during anthracycline chemotherapy. Postgrad. Med J..

[65] Skyttä T., Tuohinen S., Virtanen V., Raatikainen P., Kellokumpu-Lehtinen P.L. (2015). The concurrent use of aromatase inhibitors and radiotherapy induces echocardiographic changes in patients with breast cancer. Anticancer Res..

[66] Rahmati M., Lee S., Yon D.K., Lee S.W., Udeh R., McEvoy M., Oh H., Butler L., Keyes H., Barnett Y. (2024). Physical activity and prevention of mental health complications: An umbrella review. Neurosci. Biobehav. Rev..

[67] Haider M., Hashmi M.S.A., Raza A., Ibrahim M., Fitriyani N.L., Syafrudin M., Lee S.W. (2024). Novel Ensemble Learning Algorithm for Early Detection of Lower Back Pain Using Spinal Anomalies. Mathematics.

[68] Iqbal M.S., Naqvi R.A., Alizadehsani R., Hussain S., Moqurrab S.A., Lee S.W. (2024). An adaptive ensemble deep learning framework for reliable detection of pandemic patients. Comput. Biol. Med..

[69] Bielecka-Dabrowa A., Ebner N., Dos Santos M.R., Ishida J., Hasenfuss G., von Haehling S. (2020). Cachexia, muscle wasting, and frailty in cardiovascular disease. Eur. J. Heart Fail..

